# *RASSF1A *promoter methylation and expression analysis in normal and neoplastic kidney indicates a role in early tumorigenesis

**DOI:** 10.1186/1476-4598-6-49

**Published:** 2007-07-16

**Authors:** Inga Peters, Kristina Rehmet, Nadine Wilke, Markus A Kuczyk, Jörg Hennenlotter, Tyark Eilers, Stefan Machtens, Udo Jonas, Jürgen Serth

**Affiliations:** 1 Department of Urology, Medizinische Hochschule Hannover, Hannover, Germany; 2 Department of Forensic Medicine, Medizinische Hochschule Hannover, Germany; 3 Department of Urology, Eberhard-Karls University, Tübingen, Germany

## Abstract

**Background:**

Epigenetic silencing of the RAS association domain family 1A (*RASSF1A*) tumor suppressor gene promoter has been demonstrated in renal cell carcinoma (RCC) as a result of promoter hypermethylation. Contradictory results have been reported for *RASSF1A *methylation in normal kidney, thus it is not clear whether a significant difference between *RASSF1A *methylation in normal and tumor cells of the kidney exists. Moreover, RASSF1A expression has not been characterized in tumors or normal tissue as yet.

**Results:**

Using combined bisulfite restriction analysis (COBRA) we compared RASSF1A methylation in 90 paired tissue samples obtained from primary kidney tumors and corresponding normal tissue. Bisulfite sequence analysis was carried out using both pooled amplicons from the tumor and normal tissue groups and subclones obtained from a single tissue pair. Expression of RASSF1A was analyzed by the use of tissue arrays and immunohistochemistry. We found significantly increased methylation in tumor samples (mean methylation, 20%) compared to corresponding normal tissues (mean methylation, 11%; *P *< 0.001). Densely methylated sequences were found both in pooled and individual sequences of normal tissue. Immunohistochemical analysis revealed a significant reduced expression of RASSF1A in most of the tumor samples. Heterogeneous expression patterns of RASSF1A were detected in a subgroup of histologically normal tubular epithelia.

**Conclusion:**

Our methylation and expression data support the hypothesis that *RASSF1A *is involved in early tumorigenesis of renal cell carcinoma.

## Background

Renal cell carcinoma (RCC) accounts for 2–3% of human malignancies and is the seventh most frequent cause of tumor-dependent death among men [1,2]. The most common histological subtypes of sporadic kidney tumors are clear cell RCC (CC-RCC) and papillary tumors [3].

Tumorigenesis of CC-RCC is frequently associated with loss and/or alteration of the short arm of chromosome 3 [4]. At least two tumor suppressors are localized on 3p21-3p25, the *von Hippel-Lindau *(*VHL*) and the RAS association domain family 1A (*RASSF1A*) genes, both found to be altered in sporadic RCC. Chromosomal alterations, point mutations and epigenetic silencing have been described to affect VHL function [5-7], while *RASSF1A *has frequently been detected to undergo promoter hypermethylation and epigenetic silencing in CC-RCC [8-11].

RASSF1A is functionally involved in cell cycle control, microtubule stabilization, cellular adhesion and motility as well as apoptosis (reviewed in [12]). Therefore, depletion of RASSF1A is associated with accelerated mitotic progression and an increased risk for chromosomal defects [13-15], enhanced cellular motility [16] and with increased tumor susceptibility in knock-out mice [17].

The multifaceted function of RASSF1A as well as its frequently detected epigenetic silencing suggests an essential role in carcinogenesis. Therefore, findings regarding *RASSF1A *methylation in histopathologically normal kidney tissue isolated adjacent to tumor tissue or from autopsy samples [8,18,19] might be indicative for an involvement of *RASSF1A *in the early tumorigenesis of CC-RCC. On the other hand, significant methylation in normal tissue has not been described in other reports [9,10,20] and a quantitative study questions that significant differences in *RASSF1A *methylation can be found comparing methylation levels in CC-RCC and corresponding normal tissue samples [11].

The detection of a methylation frequency in tumor tissue matching that in normal tissue would indicate that *RASSF1A *methylation in kidney is more likely to be independent from CC-RCC tumorigenesis. Conversely, if methylation could be found in normal tissue, which expands following tumor development, a role of *RASSF1A *in early tumorigenesis is implied. Thus, both quality and quantitative extent of methylation in histopathologically normal and tumoral tissue of tumor-bearing kidney requires systematic analysis to demonstrate whether significant methylation can be found in normal cells and a substantially increased methylation can be detected within neoplastic cells. Moreover, considering that so far the presence of RASSF1A protein has been analyzed solely in fully transformed or embryonic cell lines [14,16], it is an important issue to examine whether methylation levels can be correlated to a corresponding expression of RASSF1A in normal and tumor tissues.

In the present study, we have quantitatively measured promoter methylation of the *RASSF1A *gene in paired samples of CC-RCC and corresponding normal renal tissue. Furthermore, the presence of RASSF1A protein in tumors and histopathologically normal parenchyma was analyzed using tissue arrays and immunohistochemistry. Methylation was found to be significantly increased within tumor cells of tissue pairs analyzed. Correspondingly, almost complete loss of RASSF1A expression was observed in tumor cells, while normal tubular epithelial cells largely demonstrated strong immunopositivity. However, a subgroup of tissues exhibited a heterogeneous expression pattern including partial loss of RASSF1A protein.

## Results

### Quantitation of RASSF1A methylation in paired tumoral and normal tissue samples

Methylation was first quantitatively analyzed in tumor vs. paired normal tissue from each individual patient using COBRA (Fig. 2), Fig. 3a).

**Figure 1:**
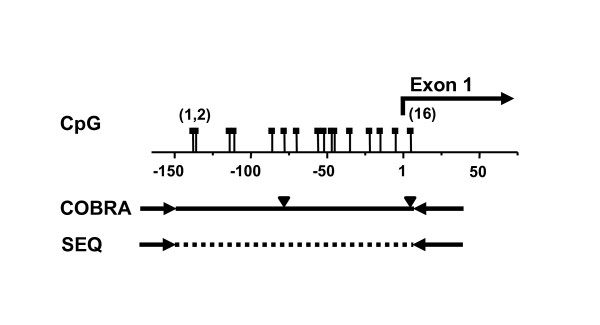
**Analyzed promoter region of the *RASSF1A *gene**. CpG sites in the promoter region of the *RASSF1A *gene analyzed by combined bisulfite restriction analysis (COBRA) and bisulfite sequence analysis. Numbers refer to the position of transcription start site while bracketed numbers indicate CpG sites. Solid triangles show TaqI restrictions sites used for COBRA. The solid and dashed lines indicate sequences amplified for COBRA and sequence analysis following bisulfite conversion of DNA. Inner primer positions were indicated by solid arrows.

**Figure 2:**
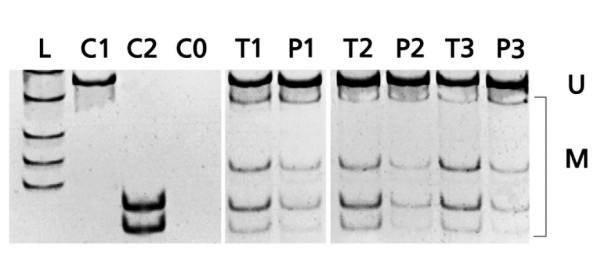
**Methylation analysis of paired tumor and normal kidney samples using COBRA**. The degree of methylation in kidney samples was analyzed using COBRA and video densitometry for determination of methylated (M) and unmethylated (U) band signals: L, length marker; C1, negative methylation control (plasmid pCU); C2, positive methylation control (plasmid pCM); CO, negative control; T1 – T3, tumoral and P1 – P3, normal paired tissue samples.

**Figure 3:**
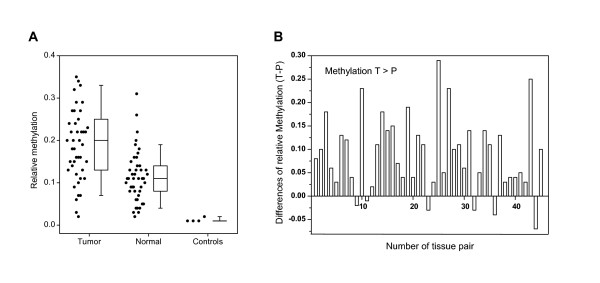
**Results of quantitative methylation analysis of tissue pairs**. a. Primary data and box plot illustration for in group comparison of relative methylation of *RASSF1A *promoter as measured by COBRA in 45 tumor and paired normal renal tissue samples as well as plasmid – DNA negative controls. Note that 44 (98%) tumors and 44 (98%) of 45 paired normal tissues demonstrated relative methylation greater than 2.75% determined as limit of analytical sensitivity. b. Quantitative methylation analysis of the *RASSF1A *promoter in 45 tumoral and normal tissue pairs using combined bisulfite restriction analysis (COBRA). Note that most of the tumors demonstrate a substantial relative increase in methylation when compared to their corresponding normal tissue (*P *< 0.001)

It is obvious from Fig. 3a that most tumors and normal tissues demonstrate relative methylation levels above the analytical detection limit, however, a relative increase of methylation in tumors when compared to the paired normal tissue was observed in 39 (86.7%) of 45 tumors (Fig. 3b). This finding is statistically significant (t-test for paired samples, P < 0.001). Note that five tissue pairs demonstrated an apparently decreased relative methylation in tumors which possibly can be deduced from small measurement variations considering that the corresponding methylation levels approximated the methodical limit of detection.

Mean degrees of methylation of 20% and 11% were determined for the tumoral and normal tissues.

### Bisulfite sequence analysis for comparison of tumoral and normal tissues

**Figure 4:**
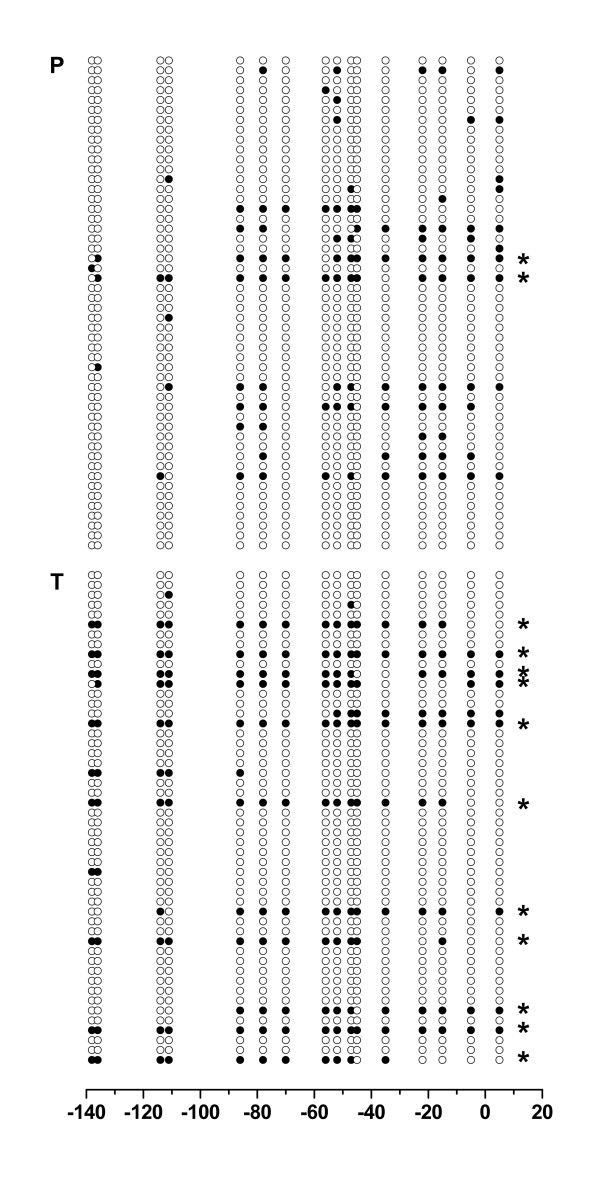
**Bisulfite sequence analysis of *RASSF1A *promoter methylation in one tissue pair of tumoral and normal tissue**. *RASSF1A *promoter amplicons obtained from a single tumoral (T) and its paired normal (P) tissue sample were subcloned and each 50 clones analyzed by the use of bisulfite sequencing. For each clone the methylation status of analyzed CpG sites is shown (solid circles: methylation, open circles: no methylation). Numbers on x-axis refer to base pair positions as indicated in Figure 1. Asterisks indicate amplicons derived from densely methylated promoters demonstrating at least 11 methylated CpG sites.

**Figure 5:**
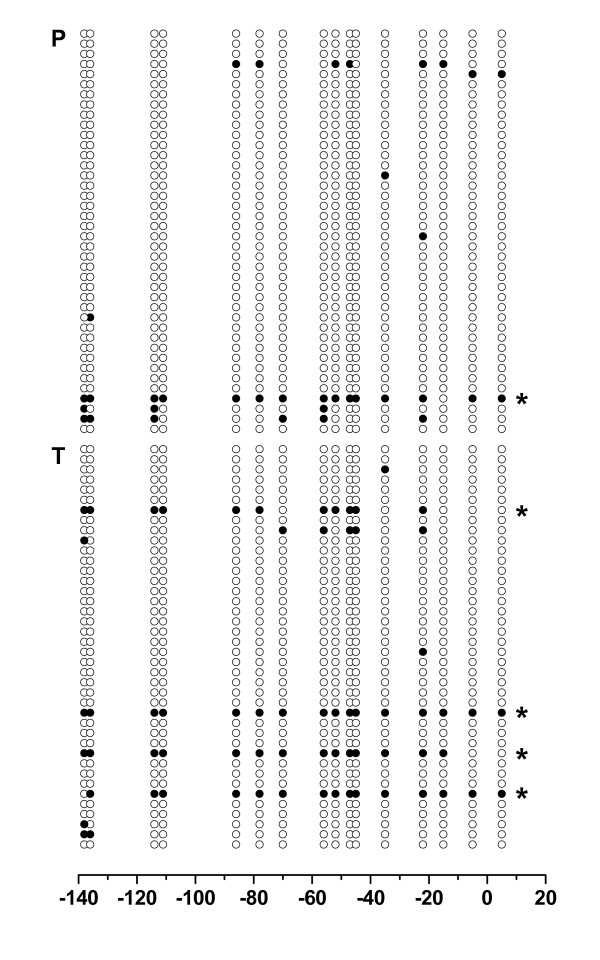
**Bisulfite sequencing of pooled tumoral and normal *RASSF1A *promoters**. *RASSF1A *promoter amplicons from 42 tumoral and 42 normal tissue samples were normalized, pooled, subcloned and each 40 subclones of the tumor (T) and normal (P) group analyzed by bisulfite sequencing. For each clone the methylation status of analyzed CpG sites is shown (solid circles: methylation, open circles: no methylation). Numbers on x-axis refer to base pair positions as indicated in Figure 1. Asterisks indicate amplicons derived from densely methylated promoters demonstrating at least 11 methylated CpG sites.

Bisulfite sequencing analysis was carried out using in each case 50 subclones obtained from an individual normal and tumoral tissue pair following bisulfite conversion and amplification (Fig. 4). We found 102 (12.8%) and 165 (20.6%) of totally analyzed 800 CpG sites to be methylated in normal and paired tumoral tissue samples, respectively (P < 0.001, chi-square test). Dense promoter methylation of more than 10 methylated CpG sites per clone was more frequently found in the tumor sample (n = 11) when compared to the paired normal tissue (n = 1; see Fig. 5).

In addition to the sequence analysis of the individual kidney tissue pair, we conducted an in-group sequence comparison of pooled amplicons obtained from the tumor and normal tissues following conversion, amplification and normalization of DNA. Thirty-five (5.5%) and 67 (10.5%) of 640 CpG sites analyzed in total were found to be methylated in the normal and tumor tissue group, respectively (Fig. 5). Assuming each CpG methylation as an independent event, statistical analysis showed that tumor and normal tissue groups demonstrated significant differences in methylation frequency (P < 0.001, chi-square test).

### Analysis of RASSF1A promoter methylation and *RASSF1A *expression in CaSki and HEK293 cell lines

**Figure 6:**
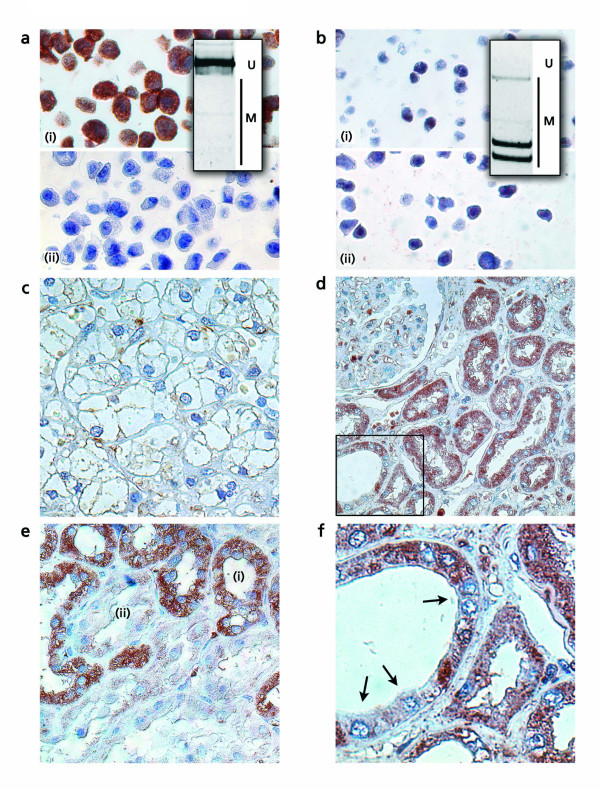
**Detection of RASSF1A protein in clear cell RCC and control cell lines**. Paraffin-embedded tissue samples obtained from renal tumor and paired normal tissues were analyzed for presence of RASSF1A protein using immunohistochemistry (counterstaining hematoxylin). a. Immunohistochemical positive control: CaSki cell line demonstrating absence of *RASSF1A *promoter methylation in COBRA (see box; U, unmethylated signals; M, methylation signals) and immunopositivity depending on the absence (*i*) or presence (*ii*) of an antibody blocking peptide, respectively. b. Immunohistochemical negative control: HEK293 cell line showing strong methylation in COBRA (see box; U, unmethylated signals; M, methylation signals). Only residual unspecific nuclear immunopositivity can be observed, which is independent from the absence (*i*) and presence (*ii*) of an antibody blocking peptide. c. Tumor cells of a clear cell RCC (magnification, 400×). d. Histopathologically normal tissue with immunopositive epithelial tubular cells (magnification, 400×). e. Heterogeneous staining patterns demonstrating immunopositive (*i*) and -negative (*ii*) epithelia in renal tubules. f. Mosaic-like staining signals in a single tubular epithelium (see arrows, magnified from panel d as indicated).

To detect RASSF1A protein in paraffin embedded tumoral and normal kidney tissue we first examined the specificity of the RASSF1A antibody using paraffin embedded sediments of control cell lines CaSki and HEK293. We found strong immunopositivity in the cytoplasm of CaSki cells (Fig. 6a[i]). This corresponds with a lack of *RASSF1A *promoter methylation as detected by COBRA (Fig. 6a). Moreover, immunopositivity can be specifically blocked by the peptide used for immunization (Fig. 6a[ii]). On the contrary, we detected almost complete methylation of *RASSF1A *in the embryonic kidney cell line HEK293, which is associated with significantly decreased cytoplasmic immunopositivity (Fig. 6b[i]). As expected this finding is not affected by peptide blocking of the antibody (Fig. 6b[ii]). Faint nuclear positivity, appears as heterogeneous pattern in antibody incubations of HEK293 independently of the presence of a blocking peptide, suggesting a residual unspecific nuclear binding of the polyclonal antibody. However, only cytoplasmic immunopositivity has been considered for evaluation of tissue sections.

### Detection of RASSF1A protein in tumoral and normal tissue samples of the kidney

Using immunohistochemistry and tissue arrays obtained from paraffin embedded renal tissue samples we analyzed 60 tissue pairs for expression of RASSF1A. We found that 33 (55%) of tumors demonstrate to a great extent loss of cytoplasmic RASSF1A protein corresponding to a labeling index of less than 10% of immunopositive stained tumor cells (Fig. 6c). In contrast, the vast majority of normal tubular epithelial cells showed strong cytoplasmic signals for RASSF1A protein (Fig. 6d). However, a subgroup of normal tissues was identified to exhibit loss of immunopositivity in some of the morphologically normal renal tubular epithelia (Fig. 6e). Moreover, a significant heterogeneity of signals appearing as a mosaic-like staining pattern was observed within single renal tubules (Fig. 6f). Note that both inter- and intratubular heterogeneous expression of RASSF1A has also been observed using immunofluorescence detection and the mouse monoclonal antibody (data not shown).

## Discussion

*RASSF1A *promoter methylation has been shown to be associated with gene silencing in RCC [8,9]. However, discordant results were reported for *RASSF1A *promoter methylation occurring in tumor-surrounding, i.e. histopathologically tumor-free renal tissue [8-10,18,20]. Moreover, a quantitative study found no difference between normal and tumoral tissue regarding the frequency of methylation or a normalized methylation index of both tissues [11]. Therefore, as long as a significant increase of tumor-dependent *RASSF1A *methylation cannot be shown in CC-RCC, it seems debatable whether *RASSF1A *substantially contributes to RCC tumorigenesis.

Our study is the first to specifically address the comparison of *RASSF1A *methylation levels detectable in paired samples of normal and tumor tissue. Our quantitative methylation analysis focused CpG sites localized directly adjacent to the start of *RASSF1A *transcription, a promoter region that has been described to cause gene silencing of *RASSF1A *[21,22]. The results of our quantitative data clearly show that the degree of relative methylation is significantly increased within tumor tissue of individual tissue pairs. However, a single tissue pair out of 45 demonstrated a 7% decrease of relative methylation in the tumor sample which we hypothetically explain by tissue heterogeneity due to the presence of undetected fibroblasts or tumor infiltrating lymphocytes.

Increased methylation as detected in most tumors can be explained by a higher proportion of low grade methylation, an increased number of highly methylated promoters or a combination of both effects. According to our bisulfite sequencing analysis of both an individual tissue pair and the tumor and normal groups as a whole, the higher degree of relative methylation observed in tumors can be attributed clearly to a higher proportion of densely methylated sequences present in tumors. Although the absolute numbers for the relative degree of methylation determined by COBRA and calculated on the basis of bisulfite sequencing slightly differ, a nearly identical ratio of methylation in tumor vs. normal tissue samples was obtained for both methods. Thus, the results of our comparative methylation analysis demonstrate a statistically significant expansion of *RASSF1A *promoter methylation in the course of RCC development.

However, our sequencing analyses also demonstrated that dense methylation, though detected more frequently in tumor cells, is also sporadically observed in normal tissue thereby confirming results described in some of the earlier analyses of *RASSF1A *methylation in kidney tissue [8,18,19]. Moreover, methylation data obtained for tumors and normal tissue overall matches the results of our expression analysis. Accordingly, expression analysis demonstrated strong signals for RASSF1A protein in the control cell line as well as in the cytoplasm of most of the normal tubular cells. In contrast, a significant loss of protein was detected within most cells of tumor tissue. Thus, protein levels detected immunohistochemically in a large part of tumors are in concordance with a gene silencing of *RASSF1A *in RCC as described previously [8-10].

Most interestingly, we also observed in normal tissue samples a partial loss of signals for RASSF1A protein, appearing as a heterogeneous staining pattern, which is in line with the detection of a subgroup of densely methylated promoter sequences in normal tissue. Similar findings have been described for methylation and expression of the *MLH1 *and *p14 *genes and were designated as "epigenetic mosaicism" [23-25].

It has been described that RASSF1A translocates during mitosis and reorganization of microtubules [14], thus leading to a loss of cytoplasmic signals. However, apparent mitotic activity was not detected in these cells, neither in extent nor in frequency explaining the observed expression patterns.

We therefore conclude that the sporadic hypermethylation observed in normal tissue corresponds to a partial loss of RASSF1A expression in normal tubular epithelium of the kidney leading to epigenetic mosaicism of *RASSF1A *as a possible premalignant event in CC-RCC tumorigenesis. To characterize the role of *RASSF1A *in early tumorigenesis of the kidney further functional studies are required.

## Conclusion

Our methylation and expression analyses of RASSF1A support the hypothesis that infrequent hypermethylation of the *RASSF1A *promoter in normal cells precedes the formation of tumor cells, thus explaining the more frequently detected hypermethylation found in most CC-RCCs. The partial loss of RASS1A expression driven by hypermethylation of the gene promoter could be a premalignant event in early tumorigenesis in the kidney.

## Methods

### Tissue samples and DNA extraction

Paired tumoral and normal specimens were prepared from 45 primary clear cell RCCs after nephrectomy (table 1 for patient characteristics). Normal tissue samples were obtained from histologically benign renal parenchyma distant to the tumor-bearing region of the kidney. All tissue samples were snap-frozen in liquid nitrogen and stored at -80°C. Following preparation of 5 *μ*m frozen sections and hematoxylin-eosin staining, two serial cuttings of each appropriate tissue – i.e. tumor tissue consisting at least of 75% tumor cells and histopathologically normal tissue – were subjected to proteinase K digestion. One section was dedicated to control quality and quantity of extracted DNA using agarose gel electrophoresis and videodensitometrical analysis. The remaining aliquot was utilized for subsequent bisulfite conversion. In summary, tissue sections were incubated on the slide using 0.5 mg proteinase K in 50 *μ*L of digestion buffer (50 mM KCl, 10 mM Tris-HCl pH 8.3, 0.5 mM EDTA, 0.01% w/v gelatin, 0.5% Tween 20) for 20 min at room temperature in a humid chamber. Digestion of tissue sections was completed after transfer of partially digested sections into a 1.5 mL reaction tube and incubation at 50°C for another 3 h. DNA was extracted using standard phenol/chloroform extraction and phase lock tubes (Light 0.5 mL, Eppendorf, Wesseling-Berzdorf, Germany) for phase separation.

**Table 1:** Pathological and clinical characterization of tumor group.

Number of patients	45
Mean, age in years	61
Males, n (%)	34 (75.5)
Females, n (%)	11 (24.5)
Pathologic Stage, n (%)	
T1	18 (40)
T2	5 (11)
T3	20 (45)
T4	2 (4)

### Bisulfite conversion of DNA

Bisulfite conversion of genomic DNA was carried out according to Herman *et al. *[26]. To precipitate the converted DNA, the conversion mixture, 800 *μ*l water, 1 *μ*g salmon sperm DNA, 20 *μ*L MgCl_2 _(1 M) and 800 *μ*L of polyethylene glycol (PEG) 1,500 was mixed by inversion, incubated for 1 h at 0°C and centrifuged for 20 min (15,000 *g*, 4°C). Finally, the precipitated DNA was dissolved and desulfonated by addition of 100 *μ*L NaOH (0.3 M) and incubation at room temperature for 15 min. Following standard ethanol precipitation the DNA was resuspended in 50 *μ*L Tris-EDTA buffer (10 mM, pH 8.0) and stored at -80°C.

### Methylation analysis

CpG sites in *RASSF1A *promoter region analyzed by combined bisulfite restriction analysis (COBRA) and bisulfite sequence analysis are shown in Figure 1. Plasmids pCM (converted methylated) and pCU (converted unmethylated), were generated by cloning amplicons of methylated and unmethylated native promoter sequence following bisulfite conversion and then applied as reference sequences for quantitative methylation analysis.

Combined bisulfite restriction analysis was carried out using semi-nested PCR and primers as described previously [21]. A uniform amplification of the converted methylated and unmethylated sequences could be maintained for copy numbers equal or greater than 5 × 10^3 ^sequence copies (data not shown). For first round amplification 5 *μ*L of converted DNA, 0.4 *μ*M of each primer, CU001 (5'-GTT TTG GTA GTT TAA TGA GTT TAG GTT TTT T-3') and CL002 (5'-ACC CTC TTC CTC TAA CAC AAT AAA ACT AAC C-3'), 1 unit *Taq *polymerase (Quiagen, Hilden, Germany), 0.2 mM dNTPs and 5.5 *μ*L of reaction buffer were mixed (total volume 50 *μ*L) and subjected to PCR (initial denaturation at 95°C for 180 sec; 25 cycles with 45 sec of denaturation at 95°C, annealing at 63°C for 1 min, primer extension at 73°C for 45 sec and 180 sec at 72°C for the last cycle). The second PCR product was generated using 1 *μ*L of first PCR product and primers CU001 and CN003 (5'-CCC CAC AAT CCC TAC ACC CAA AT-3'). PCR conditions were identical to the first PCR except an annealing temperature of 56°C was applied in a total PCR volume of 25 *μ*L. For restriction digest 10 *μ*L of the second PCR, 3.3 units of Taq I (New England Biolabs, USA), water and the corresponding restriction buffer were incubated over night at 65°C in a final volume of 20 *μ*L and subsequently analyzed using PAGE and video densitometry.

To determine the relative degree of methylation, fragments corresponding to methylated sequences and the uncut band indicating unmethylated sequences were quantified by video densitometry and corrected for molecular weight. The relative degree of methylation in each sample was calculated by dividing the sum of methylated signals by the sum of all signals. To demonstrate that COBRA can be applied for determination of the relative degree of *RASSF1A *promoter methylation, defined mixtures of corresponding plasmid DNA and of DNA isolated from HEK293 and CaSki cell lines were measured. The relative degree of methylation measured by COBRA was then compared to the known input ratio of methylated and unmethylated sequences. Linearity was observed for almost two orders of magnitude, both for the plasmid and cell line controls. Using the threefold standard deviation of signals obtained from multiple negative controls, the analytical sensitivity of COBRA could be calculated to be 2.75% relative methylation (Fig. 3a).

### Bisulfite sequencing

For sequence analysis amplicons were subcloned into pGEM-T Easy vectors (Promega, Mannheim, Germany) and sequenced using SP6 primer, BigDye Terminator v1.1 Cycle Sequencing Kit on an ABI 3100 Avant automated sequencer (Applied Biosystems, Darmstadt, Germany) according to the manufacturer's protocols or by custom sequence analysis (MWG Biotech, Ebersberg, Germany). Pool sequencing analysis was carried out following normalization of 42 amplicons obtained each from the tumor and normal tissue groups and subcloning as described above.

### Immunohistochemistry

Immunohistochemical analysis of renal paraffin embedded tissue samples arranged in an array format and signal amplification using tyramide was carried out as described previously [27-29]. For detection of RASSF1A protein an affinity purified goat polyclonal antibody (RASF1-N15, Santa Cruz Biotechnology, USA) and a mouse monoclonal antibody (Anti-human RASSF1A mAb, clone 3F3, Acris, Germany) were applied. RASF1-N15 specifically detects the N-terminus of RASSF1 isoforms A, D, F, G. However, the major transcripts are represented by isoforms A and C [21], whereas other isoforms seem to be expressed rarely in a tissue-specific manner such as RASSF1D and E [30]. Expression of RASSF isoform C has been localized to the nucleus [14], thus cytoplasmic signals obtained by immunostaining using RASF1-N15 antibody were considered to be due to RASSF1A expression.

### Statistical analysis

Statistical analyses were performed using the SPSS statistical software (statistical package for social sciences, SPSS, Inc., Chicago, IL). P-values of ≤0.05 were considered statistically significant.

## Competing interests

The author(s) declare that they have no competing interests.

## Authors' contributions

IP carried out the methylation analyses and participated in the sequence analysis and drafting of the manuscript and figures. KR participated in sequence analysis. NW and JH were engaged in isolation and characterization of tissue samples. MK and SM participated in the design of the study and data analysis. TE assisted in method development for DNA precipitation following bisulfite treatment. UJ assisted with general scientific discussion. JS conceived of the study, participated in its design, statistical analysis and coordination and wrote the original and final versions of the manuscript.

All authors read and approved the final manuscript.
